# Sliding Ferroelectric Metals: An Emerging Frontier

**DOI:** 10.1002/advs.78232

**Published:** 2026-10-14

**Authors:** Ying Yang, Zhenzhou Guo, Xia Cheng, Xiaotian Wang

**Affiliations:** ^1^ School of Physics and Optoelectronic Engineering Chongqing Normal University Chongqing People's Republic of China; ^2^ Institute For Superconducting and Electronic Materials, Faculty of Engineering and Information Sciences University of Wollongong Wollongong New South Wales Australia; ^3^ School of Physical Science and Technology Southwest University Chongqing People's Republic of China

**Keywords:** antiferromagnetism, sliding ferroelectricity, two‐dimensional metals

## Abstract

Sliding ferroelectric (FE) metals have emerged as a distinct paradigm in which interlayer sliding enables switchable out‐of‐plane polarization in a metallic state, overcoming the conventional incompatibility between FE switching and metallicity. This Perspective traces the development of this field from its foundation in nonmagnetic systems, including 1T′‐WTe_2_ and T_d_‐MoTe_2_, in which switchable polarization coexists with metallic electronic states and enables Berry curvature memory and electrically controlled superconductivity. Recent extensions to systems hosting compensated magnetic orders are also discussed. Representative material platforms further reveal that these magnetic sliding FE metals can host triply‐coupled switching, sizable linear magnetoelectric coupling, and electrically tunable anomalous transport properties. Critical challenges to translating these advances into practical applications are further identified, encompassing material discovery, experimental verification of triply‐coupled switching, and the development of spintronic devices.

## Graphical abstract

This Perspective highlights recent advances in sliding ferroelectric metals, focusing on the coexistence of switchable polarization and metallicity. Representative nonmagnetic and magnetic systems are discussed, together with Berry‐curvature memory, superconducting switching, triply‐coupled switching, magnetoelectric coupling, and electrically controllable anomalous transport.

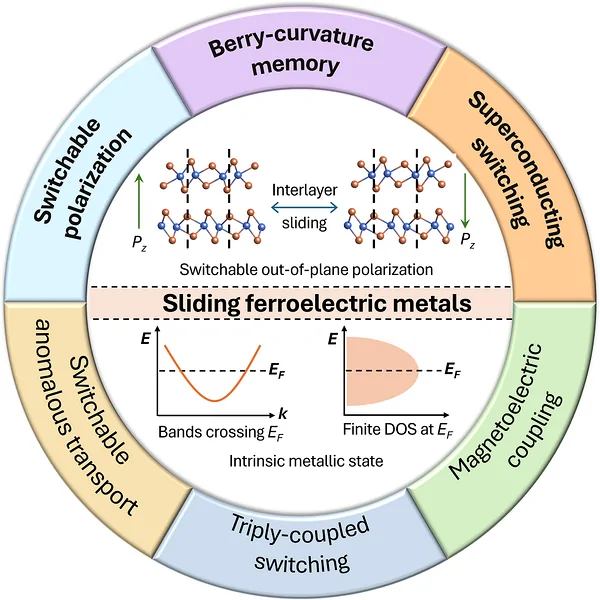

## 1 Introduction

Ferroelectric (FE) materials have garnered extensive attention owing to their reversible spontaneous electric polarization under an electric field, a feature that serves as the cornerstone for diverse technologies ranging from non‐volatile memories and logic processors to microelectromechanical systems [1, 2, 3, 4, 5, 6, 7, 8, 9, 10, 11, 12]. Nevertheless, as electronic devices advance toward greater miniaturization, the widespread integration of conventional FE materials is hindered by two fundamental bottlenecks: (1) As FE films approach the nanoscale, the depolarization field intensifies dramatically and can completely suppress FE order once a critical thickness is reached [13, 14, 15]; (2) In bulk metals, electrostatic screening by free carriers disrupts long‐range polar correlations, rendering ferroelectricity and metallicity intrinsically incompatible [16, 17].

Despite this apparent incompatibility, Anderson and Blount in 1965 proposed that a metal could undergo an inversion‐symmetry‐breaking transition into a polar phase, thereby laying the conceptual foundation for polar metals [18]. Nearly five decades later, in 2013, Shi et al. experimentally observed a ferroelectric‐like structural transition in metallic LiOsO_3_ [19]. Subsequently, Puggioni and Rondinelli identified several routes to polar metals that satisfy the weak‐coupling hypothesis between Fermi‐level electrons and inversion‐breaking phonons [20]. Since then, numerous candidate polar metals have been proposed [17, 21, 22, 23, 24, 25, 26], including distortive polar metals [27, 28, 29], extrinsic polar metals [30, 31], and interfacial polar metals [32, 33, 34]. By combining broken inversion symmetry with metallic conductivity, polar metals provide a promising platform for exploring unconventional superconductivity [35, 36], unconventional optical responses [37, 38], magnetoelectricity [39], and thermoelectricity [20]. Nevertheless, achieving electrically switchable polarization in conventional polar metals remains a fundamental challenge. According to Gauss's law, an internal electric field cannot be sustained within an ideal homogeneous metal, meaning that an externally applied electric field is screened and cannot effectively switch the polarization of a polar metal [16, 17]. Consequently, most conventional polar metals cannot be regarded as FE metals. This fundamental incompatibility restricts the development of next‐generation multifunctional devices requiring both robust polar control and metallic transport properties.

To address these limitations, recent research has focused on sliding FE materials in two‐dimensional (2D) van der Waals (vdW) multilayers [40, 41, 42, 43, 44, 45, 46, 47, 48, 49]. In these nanoscale systems, the polarization stems from stacking‐dependent symmetry breaking accompanied by interlayer charge transfer and can be efficiently reversed through electric‐field‐driven interlayer sliding between two distinct FE configurations. To date, many of these systems have already been experimentally verified, including boron nitride [40, 50, 51, 52, 53], as well as transition metal dichalcogenides such as rhombohedral‐stacked MoS_2_ and WSe_2_ [54, 55, 56, 57, 59, 60]. Furthermore, ultrafast and fatigue‐free switching has been achieved in sliding FE systems [63, 64], highlighting the great potential of 2D sliding FEs for next‐generation non‐volatile memory technologies.

More importantly, sliding ferroelectricity with out‐of‐plane polarization can also be realized in metallic systems. Despite this potential, the realization of sliding ferroelectricity in metallic systems remains extremely rare, mainly due to electrostatic screening and the difficulty of electrically driving interlayer structural switching, which requires sufficiently thin metallic layers and sufficiently low interlayer sliding barriers [17, 43, 65]. At present, only two materials have been experimentally identified, namely 1T′‐WTe_2_ and T_d_‐MoTe_2_ [65, 66]. In these materials, sliding ferroelectricity enables non‐volatile Berry curvature memory [67] and reversible transitions between superconducting and normal states [66], respectively. In addition, integrating compensated magnetic order into sliding FE metals has been theoretically predicted to achieve the simultaneous electrical reversal of FE polarization, nonrelativistic spin splitting, and net magnetization, collectively referred to as triply‐coupled switching [68, 69, 70]. This coupling further enables multifunctional properties, including sizable magnetoelectric coupling and large, electrically reversible anomalous transport and magneto‐optical responses [69, 71, 72]. These features position sliding FE metals as promising platforms for ultralow‐power topological memory, electrically reconfigurable superconducting electronics, and multifunctional spintronic and magneto‐optical devices.

In this Perspective, we review recent theoretical and experimental progress in the emerging field of sliding FE metals and their magnetic extensions. We begin by reviewing the realization of nonmagnetic sliding FE metals, with particular emphasis on the experimentally established 1T′‐WTe_2_ and T_d_‐MoTe_2_. We discuss the coexistence of electrically switchable out‐of‐plane polarization with metallic or semimetallic electronic states and the associated functionalities beyond polarization switching. Subsequently, we turn to sliding FE metals with compensated magnetic orders, highlighting triply‐coupled switching, sizable magnetoelectric coupling, and electrically controllable anomalous transport properties that arise from the unique interplay of metallicity, sliding ferroelectricity, and compensated magnetism. Finally, we identify key challenges and future directions, from expanding the material database through high‐throughput screening to experimental verification of coupled switching and the exploration of device‐oriented functionalities, with the aim of providing a roadmap for harnessing sliding FE metals in next‐generation spintronic and quantum technologies.

## 2 Nonmagnetic Sliding FE Metals

Within the metallic framework with sliding ferroelectricity, an external electric field can switch the out‐of‐plane polarization while preserving intrinsic metallic conductivity, enabling robust FE functionality [73]. Specifically, when the polar axis is oriented in the out‐of‐plane direction in an atomically thin conducting layer, the finite thickness of the system constrains the amount of free charge that can participate in screening an external electric field. Therefore, polarization reversal can be driven by an electric field applied along the relatively less conductive out‐of‐plane direction [17]. Notably, this does not mean that electrostatic screening is absent. Instead, the out‐of‐plane polarization arises from symmetry‐breaking interlayer charge redistribution and coexists with electrostatic screening. In the following, we discuss representative material realizations of sliding FE metals and their potential applications.

### 2.1 Material Realization

Following the proposal of sliding ferroelectricity in 2017 [40], sliding FE switching was soon experimentally realized in atomically thin metallic 1T′‐WTe_2_ [65]. In this material, the emergence of ferroelectricity is rooted in symmetry breaking that depends on the layer number within the distorted 1T′ lattice. The monolayer structure is centrosymmetric and nonpolar (NP), whereas the bilayer and trilayer structures break inversion symmetry (P) and develop an out‐of‐plane polar axis that is constrained by the *b*–*c* mirror and *a*–*c* glide planes (see Figure 1a). To identify and characterize ferroelectricity in this system, Fei et al. employed dual‐gated transport measurements in atomically thin 1T′‐WTe_2_ devices, in which an electrically contacted WTe_2_ flake was encapsulated by top and bottom hexagonal boron nitride dielectrics and gated by few‐layer graphene electrodes (see Figure 1b). Transport measurements directly revealed a hallmark of ferroelectricity, namely bistability under a swept electric field. As shown in Figure 1c, bilayer and trilayer 1T′‐WTe_2_ exhibit robust and reproducible conductance hysteresis under a swept perpendicular electric field from 4 K up to above room temperature, while the centrosymmetric monolayer shows no bistability. To further corroborate that the hysteresis originates from out‐of‐plane polarization, Fei et al. used monolayer graphene as the top gate and employed the graphene conductance as an electrostatic sensor, enabling highly sensitive detection of the potential change induced by FE reversal (see Figure 1d,e). By quantifying this electrostatic readout, they estimated the polarization to be approximately 1 × 10^4^ *e*/cm at 20 K, corresponding to an interlayer charge transfer of approximately 2 × 10^11^ e/cm^2^, and found that the polarization decreases gradually with increasing temperature while remaining observable near and above room temperature (see Figure 1f). More importantly, by investigating the effect of gate‐induced charge doping, Fei et al. demonstrated that this FE switching persists well into the metallic regime (see Figure 1g), where the areal density of added electrons *n_e_* exceeds the critical density *n_c_* ≈ 2 × 10^12^ cm^−2^ for metallic transport. This provides direct evidence for the coexistence of ferroelectricity and metallicity in a single phase. Notably, they further clarified that the strong sensitivity of conductance to polarization is consistent with a layer‐resolved redistribution of charge between the two 1T′‐WTe_2_ layers under a perpendicular field. Together with the comparable interlayer potential changes associated with the applied electric field and polarization reversal, these observations point to an electronic origin dominated by interlayer charge transfer rather than a conventional lattice instability. Furthermore, Sharma et al. reported room‐temperature ferroelectricity in bulk crystalline WTe_2_, where antiparallel FE domains were directly visualized by piezoresponse force microscopy [74]. They further demonstrated reversible polarization switching in approximately 15 nm thick WTe_2_ flakes under an external bias, extending the experimental evidence for ferroelectricity from few‐layer devices to thicker samples and bulk crystals.

**FIGURE 1:**
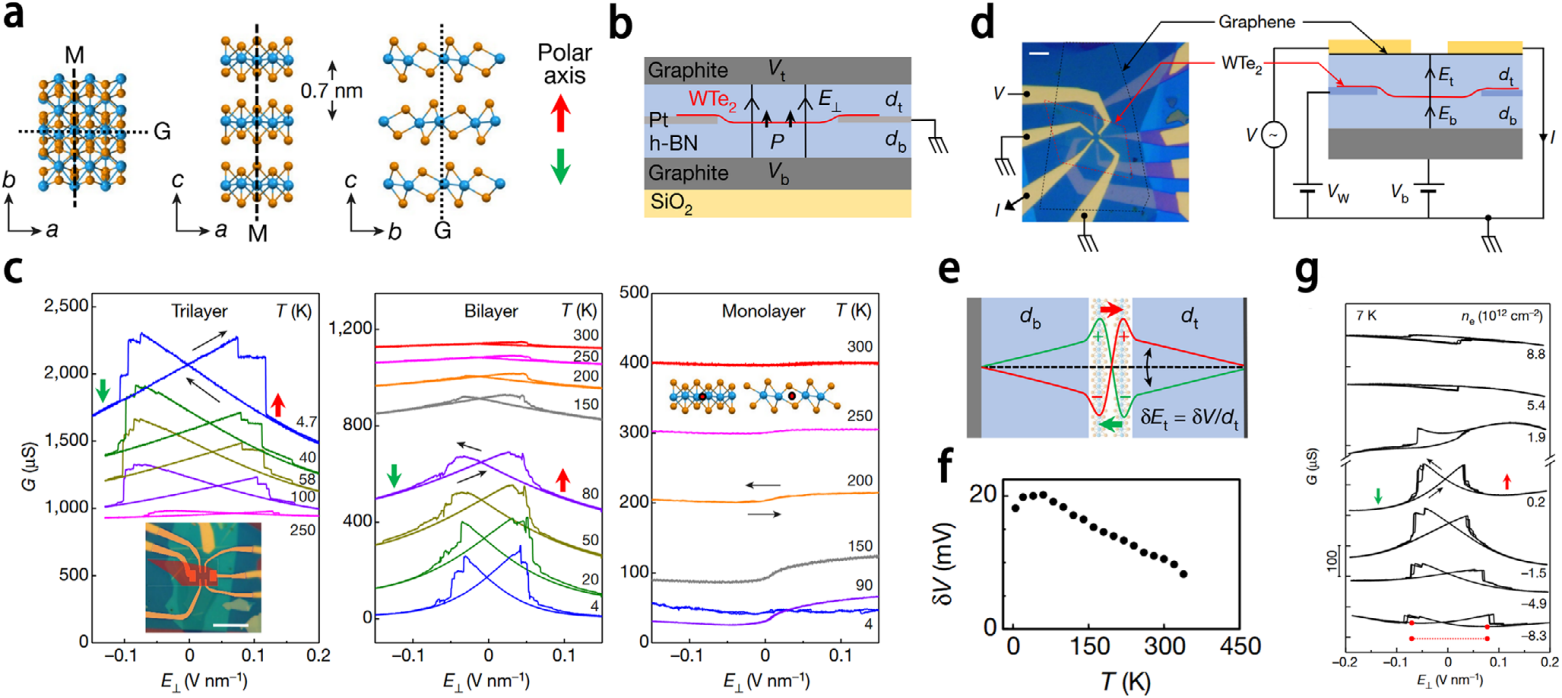
(a) Crystal structure of 1T′‐WTe_2_ illustrating the mirror plane, glide plane, and polar *c*‐axis. (b) Schematic cross‐section of the dual‐gated device used to apply a perpendicular electric field *E*_⊥_ to an atomically thin 1T′‐WTe_2_ flake. (c) Conductance *G* as a function of perpendicular electric field *E*_⊥_ for trilayer, bilayer, and monolayer 1T′‐WTe_2_ devices measured at different temperatures. (d) Micrograph (left) and schematic cross‐section (right) of a bilayer 1T′‐WTe_2_ device. (e) Schematic illustration of polarization reversal and the associated change in electrostatic potential across the device. (f) Temperature dependence of the extracted voltage shift δ*V*, which is proportional to the out‐of‐plane polarization. (g) Conductance *G* as a function of perpendicular electric field *E*_⊥_ at different carrier densities *n*_e_. Reproduced with permission [65]. Copyright 2018, Springer Nature.

From a theoretical perspective, Yang et al. demonstrated via first‐principles calculations that the reversible polarization in bilayer 1T′‐WTe_2_ originates from in‐plane sliding‐induced interlayer charge transfer [73]. As shown in Figure 2a, state I and state II of bilayer 1T′‐WTe_2_ are related by the mirror symmetry $M_{z}$ (reflection across the central horizontal plane), and therefore constitute two FE states with opposite out‐of‐plane polarizations. Furthermore, state II can be reached from state I by sliding the upper layer along the −x direction by a distance of **𝑎** + **𝑏** (0.72 Å). The FE switching between these two states follows this interlayer sliding pathway with a very small energy barrier of roughly 0.6 meV per unit cell (see Figure 2a). These FE states exhibit an out‐of‐plane polarization of approximately 3.2 × 10^11^ *e*/cm^2^, which is comparable in magnitude to the experimental value of about 2 × 10^11^ *e*/cm^2^ measured at 20 K. They attributed this out‐of‐plane polarization to the nonequivalence of the two layers in the FE states, which gives rise to an uncompensated interlayer charge transfer. For multilayer 1T′‐WTe_2_, the FE switching is likewise governed by relative in‐plane sliding between selected layers. As shown in Figure 2b, the polarization reversal is realized by sliding the middle layer by **𝑎** + **𝑏** in the trilayer and sliding the second and fourth layers by **𝑎** + **𝑏** simultaneously in the five‐layer structure. Additionally, they proposed that a small twist angle between the two layers in bilayer 1T′‐WTe_2_ can generate FE domains with opposite polarization orientations (see Figure 2c).

**FIGURE 2:**
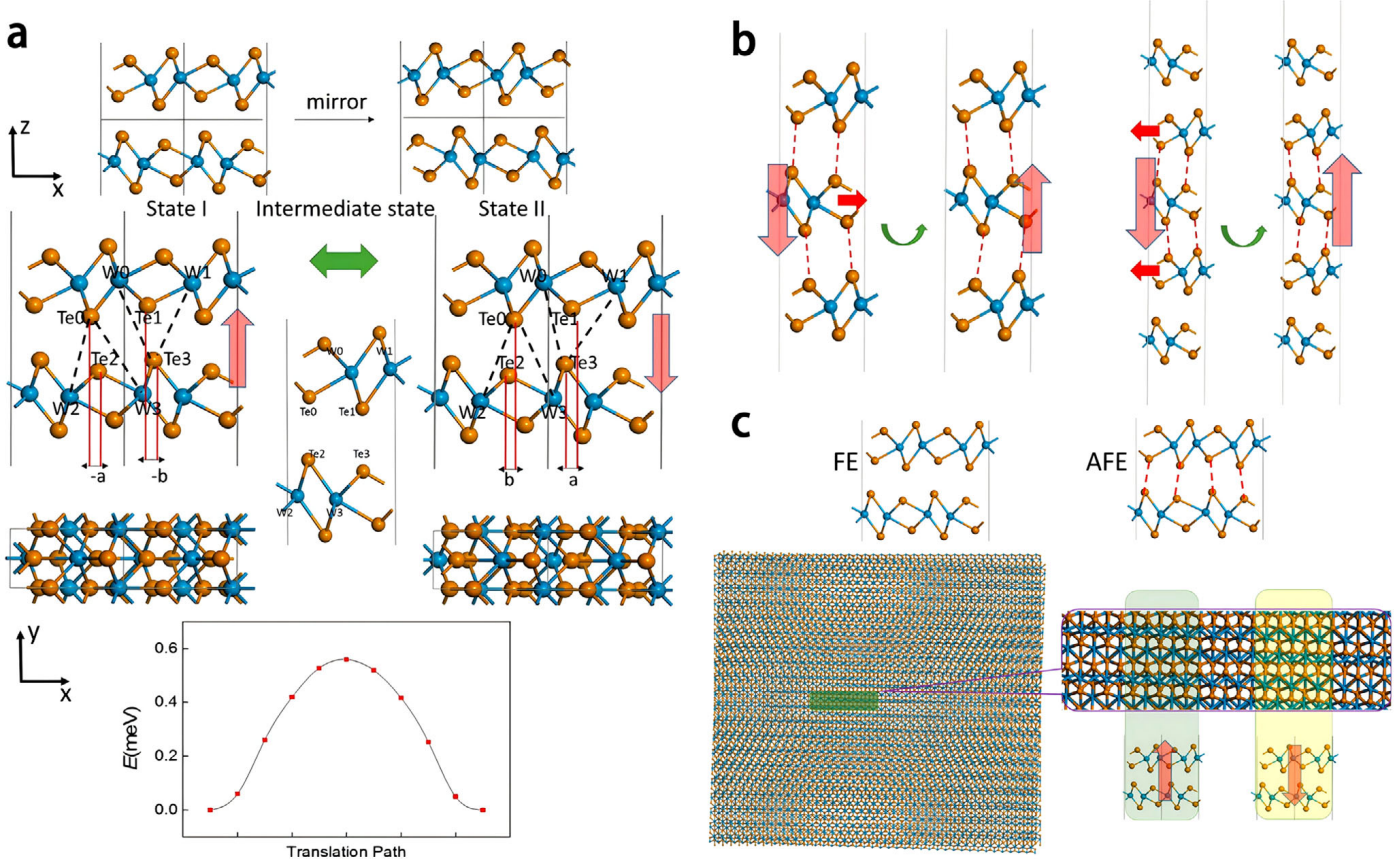
(a) The geometric structure of bilayer 1T′‐WTe_2_ states I and II obtained via interlayer translation (upper panel), and the FE switching pathway from state I to state II in bilayer 1T′‐WTe_2_ films (lower panel). (b) FE switching of 1T′‐WTe_2_ trilayer and five‐layer structures. (c) Geometric structure of FE and artificial antiferroelectric configuration (upper panel), and FE domains formed by moiré patterns upon a small angle twist of bilayer 1T′‐WTe_2_ (lower panel). Reproduced with permission [73]. Copyright 2018, American Chemical Society.

Building on the transport‐based switching signatures and the sliding‐based microscopic mechanism established earlier, de la Barrera et al. directly quantified the spontaneous polarization of bilayer 1T′‐WTe_2_ in the metallic state by employing capacitive sensing in dual‐gated mesoscopic devices [75]. By measuring the capacitance *C*_t_ between the top gate and the bilayer (Figure 3a) and sweeping the perpendicular electric field back and forth at fixed carrier density, they observed a clear switching of the polar orientation. As shown in Figure 3b, *C*_t_ exhibits abrupt changes across the full density range. When the sweep proceeds toward positive *E*_⊥_, the jump occurs at a positive critical field $E_{C}^{+}$, whereas reversing the sweep shifts the transition to a negative critical field $E_{C}^{-}$. Based on the capacitance difference Δ*C*_t_ between two representative sweeps taken in opposite directions, the switching critical fields generally lie within 0.1 V nm^−1^. This yields a pronounced hysteresis loop at each carrier density (see Figure 3c), providing direct evidence of FE switching and the associated polarization bistability in the bilayer 1T′‐WTe_2_. More importantly, owing to the gate‐tunable electron and hole densities in the bilayer 1T′‐WTe_2_ system, they constructed a polarization–doping map that tracks the evolution of out‐of‐plane polarization with electrostatic doping (see Figure 3d). The results indicate that the polarization is screened far more strongly under hole doping than under electron doping at comparable carrier densities. As holes are added, the polarization decreases rapidly and falls to nearly zero, entering a screened polar metal regime. In marked contrast, electron doping leads to a much slower decay, allowing a sizable polarization to persist deep into the sustained FE n‐type metal regime up to high electron densities. This behavior provides clear quantitative evidence for the robust coexistence of ferroelectricity and metallicity over a wide density range within a single device. More recently, Bai et al. demonstrated sub‐nanosecond polarization switching in 1T′‐WTe_2_, with a characteristic switching time down to approximately 0.6 ns and no fatigue after 10^10^ switching cycles, highlighting the potential of sliding FE metals for ultrafast and high‐endurance electronic applications [76].

**FIGURE 3:**
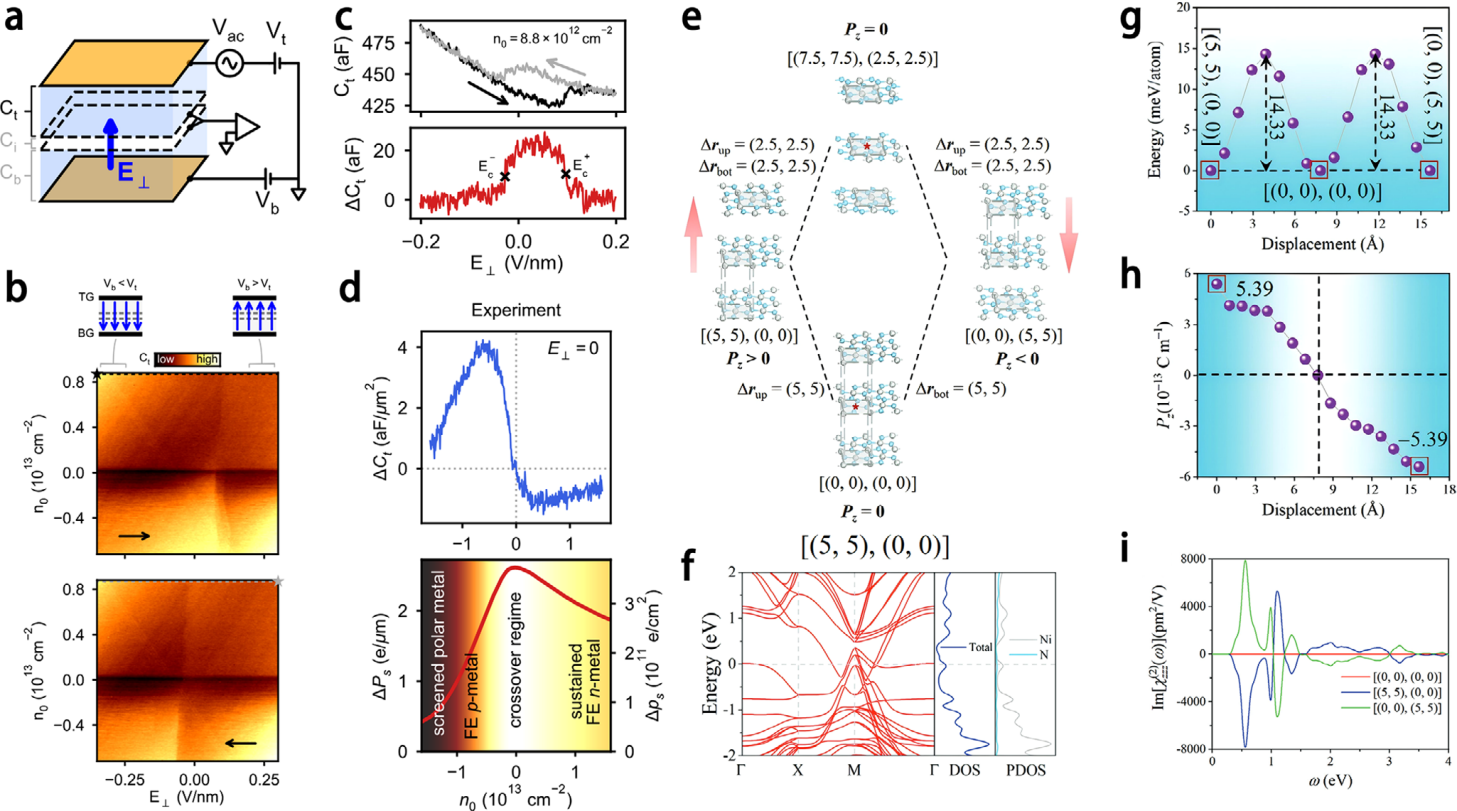
(a) Schematic illustration of the capacitance measurement used to probe out‐of‐plane polarization in atomically thin WTe_2_ devices. (b) Capacitance mapping as a function of electric field and carrier density. (c) Upper panel: capacitance traces measured along the dashed lines in (b). Lower panel: the field‐induced change in capacitance associated with polarization switching. (d) Experimental capacitance response Δ*C*_t_ at *E*_⊥_ = 0 (upper panel) and change in measured spontaneous polarization as a function of carrier density (lower panel), illustrating the crossover between screened polar metal, FE polar metal, and sustained FE metal regimes. Reproduced with permission [75]. Copyright 2021, The Authors, Published by Springer Nature. (e) The sliding patterns of the trilayer penta‐NiN_2_ with their spontaneous polarization along the out‐of‐plane direction. (f) Electronic band structures, density of states (DOS), and partial DOS of the FE phase of the trilayer penta‐NiN_2_. (g) Transition energy barriers and (h) spontaneous polarization as a function of interlayer displacement of the trilayer penta‐NiN_2_. (i) Imaginary part of the SHG susceptibilities Im[χ(2) zzz(ω)] of the trilayer penta‐NiN_2_ with the [(0, 0), (0, 0)], [(5, 5), (0, 0)], and [(0, 0), (5, 5)] stacking configurations. Reproduced with permission [77]. Copyright 2025, Wiley‐VCH GmbH.

Beyond the 1T′‐WTe_2_ system, similar nonmagnetic sliding FE metallic behavior has been reported in bilayer T_d_‐MoTe_2_, which will be discussed in detail in the next section, and theoretically predicted in trilayer penta‐NiN_2_ [77] (see Figure 3e–i). Because the monolayer penta‐NiN_2_ possesses high symmetry in the P4/mbm (No. 63) layer group, any bilayer stacking obtained by interlayer sliding remains centrosymmetric and therefore an NP state. In contrast, in the trilayer penta‐NiN_2_, two degenerate polar stackings with opposite out‐of‐plane polarization emerge when either the top or the bottom layer is laterally shifted relative to a fixed middle layer, whereas the AAA‐like configuration serves as a centrosymmetric NP state. The indirect sliding FE path yields a low switching barrier of 14.33 meV per atom (see Figure 3g) and a reversible out‐of‐plane polarization of ± 5.39 × 10^−13^ C/m (see Figure 3h). The FE phases are intrinsically metallic, with bands crossing the Fermi level (see Figure 3f), confirming trilayer penta‐NiN_2_ as a sliding FE metal. More importantly, Hou et al. further showed that second harmonic generation (SHG) can clearly distinguish the three stackings (see Figure 3i). The centrosymmetric NP state is SHG silent, whereas the two polar states exhibit SHG responses with equal magnitude but opposite sign, thereby offering a feasible route to verify the sliding FE metal phase transition.

It is important to distinguish between intrinsically polar layered compounds and artificially stacked van der Waals systems. Metallic sliding FE phases have been experimentally demonstrated in the former, whereas experimental realizations of the latter have thus far been limited to insulating systems. At present, artificially stacked van der Waals systems combining metallicity with electrically switchable polarization remain theoretical proposals. To provide a comprehensive overview of the current material landscape, we summarize the currently reported sliding FE metals, together with their thickness, polarization, energy barrier, magnetic state, year, and corresponding references, as shown in Table 1. In conclusion, these studies establish a prototypical paradigm for sliding FE metals, in which electrically switchable polarization coexists with metallic properties and is governed by interlayer charge transfer, thereby highlighting its promise for electrically controllable functionalities. In the following, we explore the application opportunities enabled by the coupling between metallicity and ferroelectricity in these systems.

**TABLE 1:** Summary of sliding FE metals and their FE and magnetic properties.

Materials	Thickness	Polarization	Energy barrier	Magnetism	Year^refs^
WTe_2_	Bilayer/Trilayer	2 × 10^11^ e/cm^2^	—	Nonmagnetism	2018 [65]
WTe_2_	Bilayer	5.1 × 10^−2^ µC/cm^2^	0.6 meV/f.u.	Nonmagnetism	2018 [73]
T_d_‐MoTe_2_	Bilayer	—	—	Nonmagnetism	2023 [66]
PtTe_2_	Bilayer	0.46 pC/m	56 meV/u.c.	Nonmagnetism	2023 [26]
PdTe_2_	Bilayer	0.06 pC/m	32 meV/u.c.	Nonmagnetism	2023 [26]
NiTe_2_	Bilayer	0.52 pC/m	22 meV/u.c.	Nonmagnetism	2023 [26]
Hf_2_S	Bilayer	>2 pC/m	125 meV/f.u.	Ferrimagnetism	2024 [71]
Cr_0.88_Mn_0.12_I_3_	Bilayer	—	—	Ferromagnetism	2024 [78]
FeSe	Bilayer	0.18 pC/m	0.011 eV/f.u.	Ferromagnetism	2024 [79]
H‐MnTe_2_	Bilayer	0.14 pC/m	0.026 eV/f.u.	Ferrimagnetism	2024 [79]
Fe_3_GeTe_2_	Bilayer	8.3 × 10^−4^ eÅ/u.c.	13 meV/u.c.	Ferromagnetism	2024 [80]
penta‐NiN_2_	Trilayer	5.39 × 10^−13^ C/m	14.33 meV/atom	Nonmagnetism	2025 [77]
1T‐CrTe_2_	Bilayer	0.20 pC/m	≈10 meV/u.c.	Ferrimagnetism	2025 [68]
1T‐NbTe_2_	Bilayer	0.34 pC/m	15.5 meV/f.u.	Ferrimagnetism	2025 [72]
Fe_5_GeTe_2_	Bilayer	0.087 pC/m	15.4 meV/u.c.	Ferrimagnetism	2026 [69]
MnIn_2_Se_4_	Bilayer	0.37 pC/m	31.7 meV/u.c.	Ferrimagnetism	2026 [70]

### 2.2 Application in Berry Curvature Memory and Superconducting Electronics

In conventional polar metals, free carriers typically screen the electric field required for spontaneous polarization switching, making electrical readout difficult. By contrast, the coexistence of metallicity and sliding ferroelectricity opens a new paradigm for non‐volatile memory devices. In the pioneering work, Xiao et al. identified and characterized Berry curvature memory through electrically driven stacking transitions in atomically thin 1T′‐WTe_2_ devices [67]. As illustrated in Figure 4a, the polar T_d,↑_ and T_d,↓_ stackings of 1T′‐WTe_2_ host opposite momentum space distributions of Berry curvature hotspots. This makes clear that electrically driven interlayer sliding is not merely a structural rearrangement, but a direct handle for switching the sign and distribution of the Berry curvature dipole. To verify the existence of this sliding FE switching process, Xiao et al. performed in situ gate‐dependent SHG measurements under an applied electric field, which revealed a pronounced butterfly‐shaped hysteresis in both four‐layer and five‐layer samples, confirming reversible switching between the two opposite FE polar states (see Figure 4b). Moreover, to probe the influence of such switching on electronic topology, the authors measured the nonlinear Hall response (see Figure 4c), which serves as a direct indicator of the Berry curvature dipole. In trilayer 1T′‐WTe_2_, the nonlinear Hall signal exhibits a sign reversal concomitant with the sliding FE switching, reflecting an inversion of the Berry curvature distribution in momentum space and a binary memory‐switching property (see Figure 4d). By contrast, in four‐layer samples, the nonlinear Hall hysteresis signal maintains its sign despite the structural transition, highlighting a layer‐parity‐selective memory effect where Berry curvature reversal occurs exclusively in odd‐layer crystals (see Figure 4e). The pronounced odd‐even contrast arises from the distinct symmetry operations relating the T_d,↑_ and T_d,↓_ stackings in different layer numbers. Specifically, the two polar states are connected by inversion in odd‐layer WTe_2_, but by an *ab*‐plane mirror operation combined with a global half‐unit‐cell translation along the a axis in even‐layer 1T′‐WTe_2_. Owing to the pseudovector nature of out‐of‐plane Berry curvature, this difference causes the Berry curvature dipole and hence the nonlinear Hall signal to reverse sign only in odd layers, while remaining unchanged in even layers. This layer‐parity‐dependent sign switching was further confirmed by first‐principles calculations, as shown in Figure 4f,g, which reproduced the reversal of the Berry curvature dipole in odd layers and its preservation in even layers. Furthermore, this sliding‐induced topological memory exhibits exceptional energy efficiency. Due to the ultralow energy barrier for interlayer sliding (a few meV per unit cell), the estimated energy consumption for a single writing operation is as low as ∼0.1 aJ nm^−^^2^, significantly outperforming traditional phase‐change memories. Taken together, this work demonstrates that electrically driven interlayer sliding in 1T′‐WTe_2_ can convert a sliding FE metal into an ultralow‐energy, non‐volatile topological memory platform, where binary information is encoded in the Berry curvature dipole, thereby highlighting a practical route to make sliding FE metals functionally useful for future memory technologies.

**FIGURE 4:**
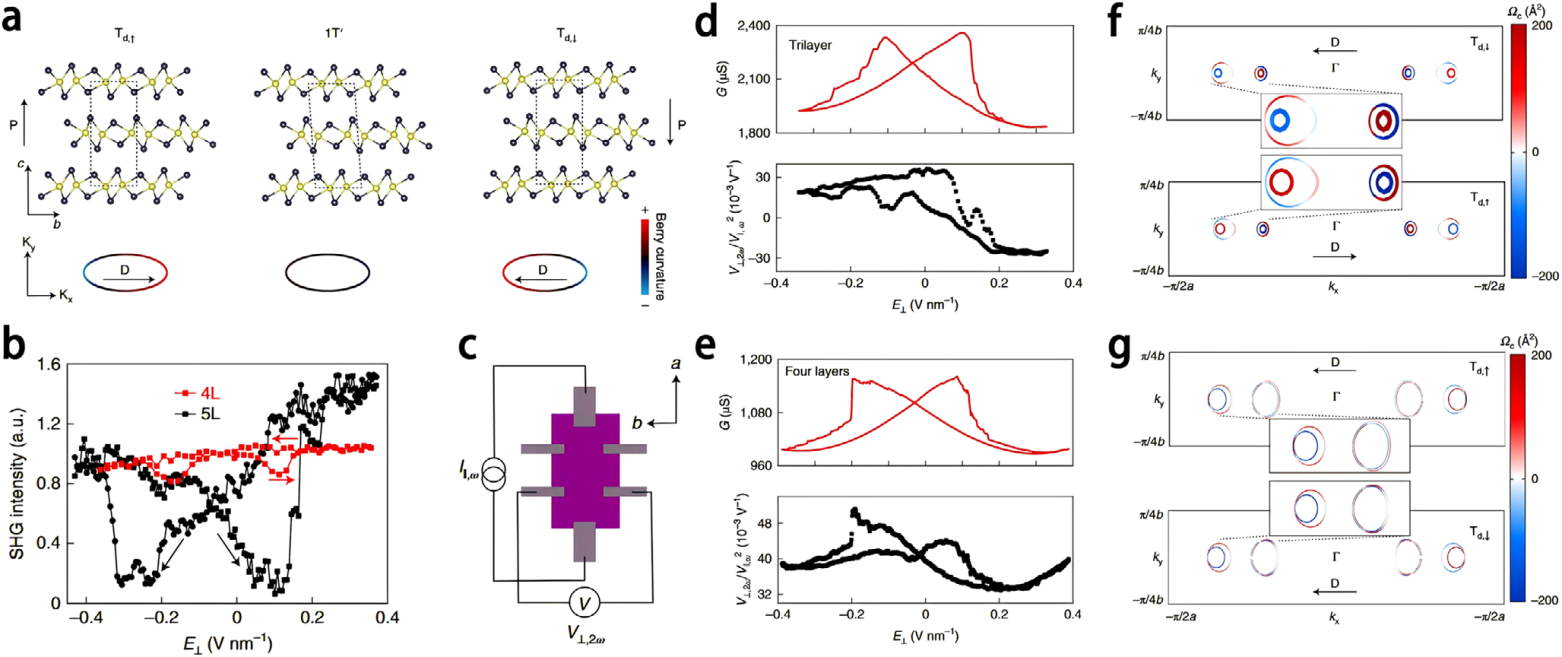
(a) Crystal structures of multilayer 1T′‐WTe_2_ illustrating different stacking configurations associated with opposite out‐of‐plane polarizations and schematics of their Berry curvature distributions in momentum space. (b) Electric‐field‐dependent SHG intensity of four‐layer and five‐layer 1T′‐WTe_2_, showing hysteretic switching between opposite polar stacking states. (c) The nonlinear Hall effect measurement schematic. (d, e) Electric‐field‐dependent longitudinal conductance (upper plot) and nonlinear Hall signal (lower plot) in trilayer 1T′‐WTe_2_ and four‐layer 1T′‐WTe_2_, respectively. (f, g) Calculated Berry curvature distribution in the 2D Brillouin zone at the Fermi level for T_d,↑_ and T_d,↓_ in trilayer and four‐layer 1T′‐WTe_2_, respectively. Reproduced with permission [67]. Copyright 2020, Springer Nature.

Beyond non‐volatile memory applications, the ability to manipulate quantum phases via a sliding FE metal opens avenues for superconducting electronics. In 2023, Jindal et al. demonstrated coupled ferroelectricity and superconductivity in atomically thin bilayer T_d_‐MoTe_2_, which exhibits compensated semimetallic behavior [66]. They employed a device in which bilayer T_d_‐MoTe_2_ samples were encapsulated by hexagonal boron nitride dielectrics and gated by graphene electrodes, allowing independent control of carrier density and displacement field (see Figure 5a). In addition, they revealed the compensated semimetallic nature of the system, where density functional theory (DFT) calculations displayed nearly compensated electron and hole pockets at the Fermi level, providing the metallic foundation for superconductivity (see Figure 5b). They then independently established the existence of superconductivity and sliding ferroelectricity in bilayer T_d_‐MoTe_2_. As illustrated in Figure 5c, bilayer T_d_‐MoTe_2_ exhibits intrinsic 2D superconductivity with a zero displacement field transition temperature of about 2.3 K, confirming the emergence of superconductivity in this atomically thin metallic system. Meanwhile, the hysteretic resistance switching under a displacement field in the normal state (4 K) reveals the coexistence of sliding ferroelectricity, indicating a reversible interlayer sliding transition between two opposite polar states in bilayer T_d_‐MoTe_2_ (see Figure 5d). Moreover, once the temperature is lowered into the superconducting regime, the electrically driven sliding FE switching becomes directly coupled to the superconducting state itself. As shown in Figure 5e, the resistance exhibits a pronounced butterfly‐shaped hysteresis under displacement field cycling at 1.7 K, where the sample switches reversibly between superconducting and normal states at the FE transition. Pulse measurements further confirmed this coupled switching, with electric field pulses of opposite polarities repeatedly driving the device between the superconducting and normal states (see Figure 5f). Once the switching was established, the sample resistance remained in the newly reached state over time. Furthermore, displacement field sweeps at different temperatures show that *T*_c_ increases continuously as the displacement field approaches the switching point and reaches its maximum immediately before polarization reversal, beyond which superconductivity is lost (see Figure 5g). This indicates that the superconducting state is intimately tied to the internal electric field of the sample.

**FIGURE 5:**
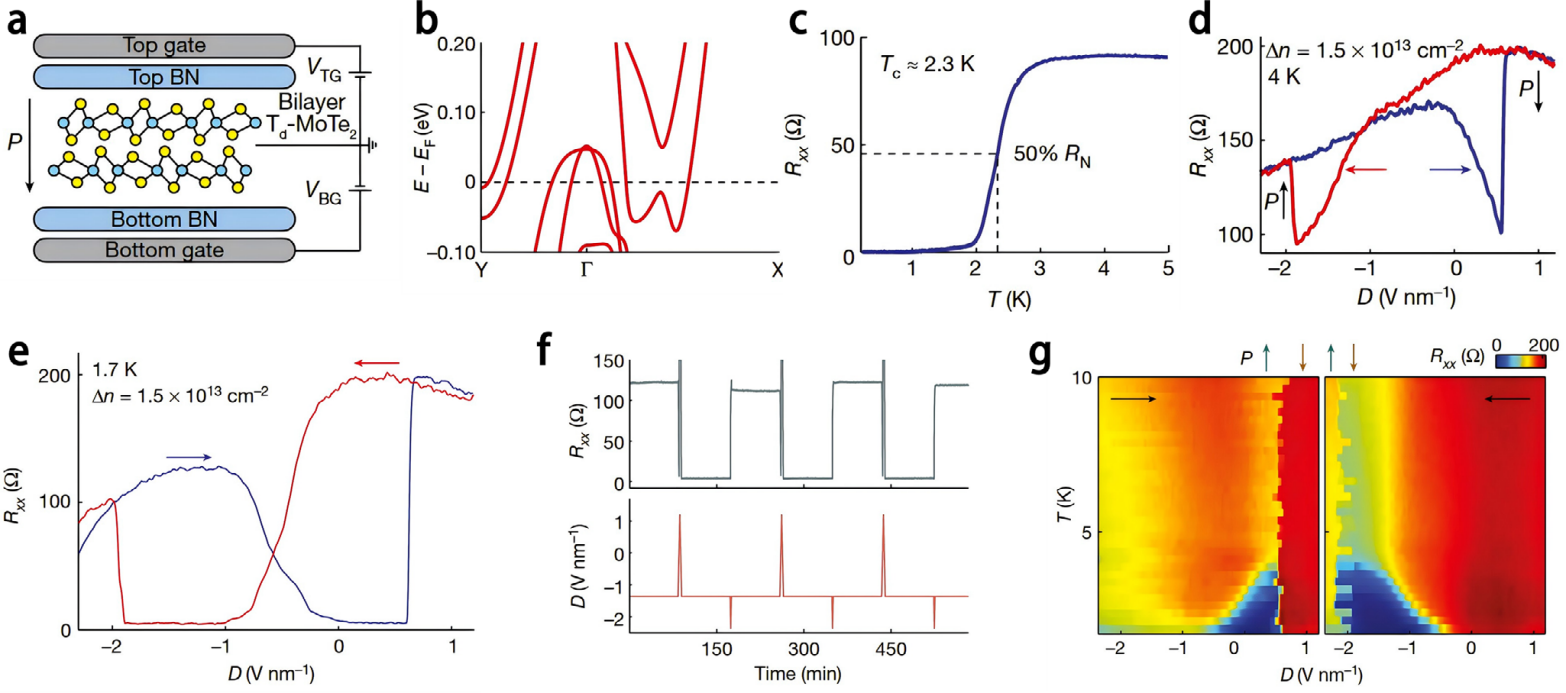
(a) Schematic of the dual‐gated device structure used to control the out‐of‐plane polarization in bilayer T_d_‐MoTe_2_. (b) DFT‐calculated electronic band structure for bilayer T_d_‐MoTe_2_. (c) Temperature dependence of the resistance *R*_xx_, showing a superconducting transition at *T*_c_ ≈ 2.3 K. (d) Electric‐field‐driven hysteretic switching at 4 K, corresponding to reversal of the FE polarization. (e) Butterfly loops with bistable normal and superconducting states, indicating coupled FE and superconducting states. (f) Reversible switching of *R*_xx_ induced by alternating displacement field pulses as a function of time. (g) Color map of *R*_xx_ as a function of the displacement field and temperature, revealing the electrically tunable superconducting regime. Reproduced with permission [66]. Copyright 2023, Springer Nature.

Taken together, these studies establish that nonmagnetic sliding FE metals are far more than unusual polar conductors. Instead, they provide a versatile materials platform in which electrically driven interlayer sliding can directly modulate functional electronic states and responses in ways that are inaccessible in conventional FEs or ordinary metals. In atomically thin 1T′‐WTe_2_, sliding FE switching enables non‐volatile Berry curvature memory by converting stacking order into a robust topological transport readout. In bilayer T_d_‐MoTe_2_, the same sliding degree of freedom becomes coupled to a collective quantum phase and allows reversible electrical switching between superconducting and normal states. These results clearly show that the central utility of nonmagnetic sliding FE metals lies not only in switchable polarization itself, but more importantly in their ability to transduce structural sliding into programmable electronic and quantum functionalities. Sliding FE metals open a promising avenue toward ultralow‐energy memory, electrically reconfigurable superconducting electronics, and broader device concepts based on the intimate interplay among stacking order, internal electric field, and metallic electronic structure.

## 3 Magnetic Sliding FE Metals

Recently, compensated magnetic orders, such as altermagnetism [81, 82, 83, 84, 85] and fully compensated ferrimagnetism [86, 87, 88, 89], have garnered intense interest as promising platforms for next‐generation spintronic devices. These compensated magnetic orders can be distinguished by the symmetry relations between their opposite‐spin sublattices in the absence of spin–orbit coupling (SOC). In conventional antiferromagnets, inversion or translation symmetry (or both) connects opposite‐spin sublattices and enforces spin‐degenerate bands [90, 91, 92, 93], whereas rotational or mirror symmetries relating the opposite‐spin sublattices give rise to the momentum‐dependent nonrelativistic spin splitting characteristic of altermagnets [94, 95]. Without symmetry relating the opposite‐spin sublattices, ferromagnet‐like spin splitting emerges, while equal spin‐channel fillings in an insulating or half‐metallic state realize filling‐enforced fully compensated ferrimagnetism [86, 96, 97, 98]. Compared with conventional ferromagnets, these compensated magnetic systems exhibit ultrafast spin dynamics in the terahertz regime and vanishing net magnetization, which provides robustness against external magnetic perturbations and enables high‐density integration without stray‐field interference [99, 100, 101, 102].

Earlier studies have extended this symmetry framework to sliding FE magnets, primarily in topologically trivial insulating and semiconducting systems [103, 104, 105, 106, 107, 108, 109, 110], where polarization reversal can switch nonrelativistic spin splitting while often preserving net magnetization. Notably, sliding FE semiconductors derived from compensated magnetic states can acquire a small uncompensated moment through spin‐dependent interfacial charge transfer. In the semiconducting MnSe bilayer, Liu et al. first predicted the simultaneous reversal of polarization, spin splitting, and a small net magnetization, with interlayer Mn‐to‐Se charge transfer producing a small net moment of 8 mµ_B_ per unit cell [107]. In addition, Huang et al. predicted a ferromagnetic sliding FE bilayer Cr_0.88_Mn_0.12_I_3_ that is metallic and hosts antiskyrmions whose spin texture can be switched by polarization reversal [78]. Building on these established foundations, subsequent studies of magnetic sliding FE metals have expanded the accessible functionality to triply‐coupled switching with sizable net magnetization, strong magnetoelectric coupling, and considerable sign‐reversible anomalous transport responses.

### 3.1 Polar‐Stacking‐Induced Ferrimagnetism in a Sliding FE Metal

Polar stacking offers an alternative pathway to magnetic sliding FE metals, where interlayer charge redistribution not only produces a switchable electric dipole but also reshapes the magnetic state of a metallic bilayer. A representative example was reported by Gui et al. in the electride bilayer Hf_2_S [71]. As shown in Figure 6a,b, polar stacking breaks P and produces an unexpectedly large out‐of‐plane polarization exceeding 2 pC/m, which is closely associated with the redistribution of delocalized interlayer anionic electrons. They further clarified that the electronic states near the Fermi level are predominantly localized at the top and bottom surface regions (see Figure 6c), whereas the FE polarization mainly originates from charge redistribution near the vdW gap. This spatial separation may help weaken screening and thereby support the coexistence of metallicity and polarization. At the same time, the surface Hf atoms experience inequivalent crystal field environments, which split the Hf 5*d* orbitals into subsets with different occupations. This orbital inequivalence induces a Stoner instability primarily at the surface layers and produces a net magnetization of about 0.35 µ_B_ per formula unit, thereby yielding a FiM state, as reflected in Figure 6d. The sliding FE switching path further reveals a direct coupling between polarization and net magnetization. Starting from one polar FiM state, interlayer sliding continuously reduces the polarization while simultaneously suppressing the net magnetic moment. After passing through the intermediate configuration, both quantities reemerge with opposite signs in the opposite polar state (see Figure 6e). Such strong intrinsic coupling allows efficient electrical control of magnetization without relying on SOC.

**FIGURE 6:**
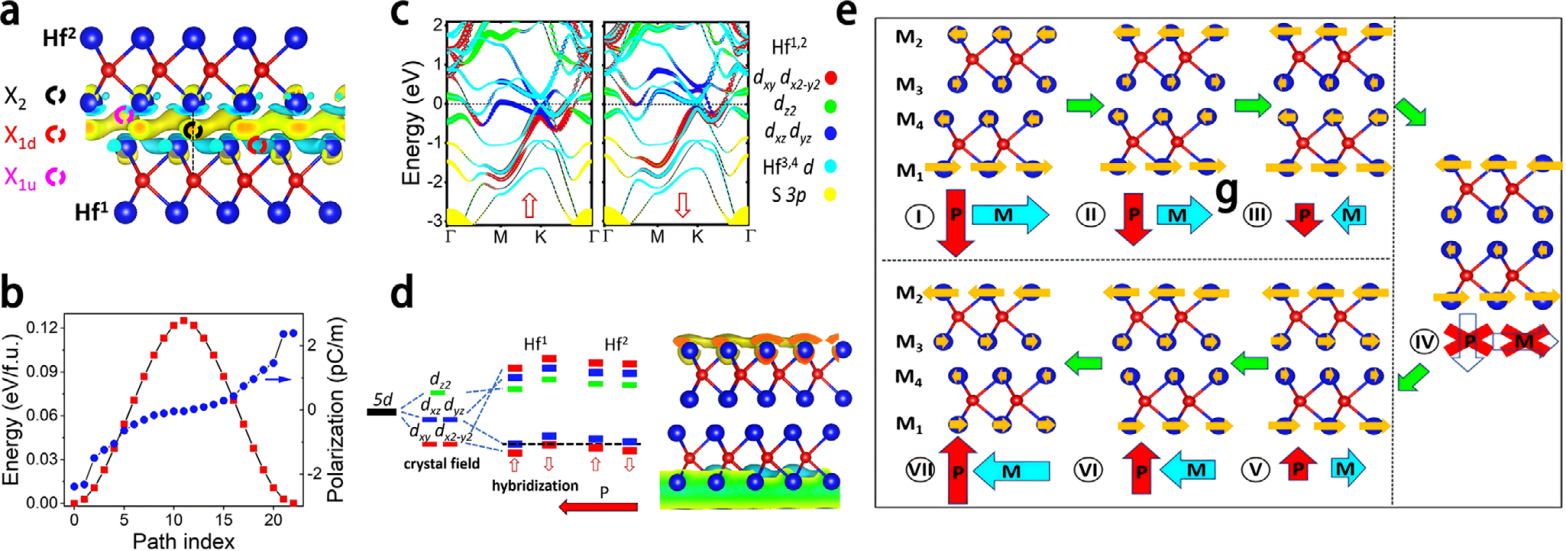
(a) Side view of the crystal structure of bilayer Hf_2_S and calculated charge density distribution of interlayer electride states. (b) Calculated energy barrier and out‐of‐plane polarization evolution along the interlayer sliding path. (c) The projected spin‐polarized electronic band structure of two opposite FE states of bilayer Hf_2_S. (d) Schematic of Hf 5*d* orbitals’ splitting due to crystal field, band hybridization, and exchange interaction (left panel) and isosurface of the magnetic charge density (right panel). (e) FE switching pathways illustrating the coupled evolution of out‐of‐plane polarization and magnetization under interlayer sliding. Reproduced with permission [71]. Copyright 2024, Springer Nature.

### 3.2 Symmetry‐Guided Design and Material Realization of Sliding FE FiM Metals

Within sliding FE metals, the simultaneous reversal of polarization, spin splitting, and net magnetization was first explored by Mavani et al. in a polar‐stacked bilayer 1T‐CrTe_2_. The system retains its metallic character and exhibits A‐type antiferromagnetic (AFM) order, with ferromagnetic (FM) intralayer coupling and AFM interlayer coupling. Opposite lateral translations from the unstable high‐symmetry NP configuration produce two energetically degenerate polar states. By breaking the symmetry relating the two magnetic layers, polar stacking generates nonrelativistic spin splitting and a small uncompensated moment of approximately 0.03 µ_B_ per Cr atom, both of which reverse together with the out‐of‐plane polarization [68].

Around the same time, our work established a general symmetry‐based route to sliding FE FiM metals with triply‐coupled switching, enabling simultaneous switching of out‐of‐plane polarization, spin splitting, and net magnetization through interlayer sliding [69]. Specifically, in sliding FE systems, the generation of out‐of‐plane polarization is primarily driven by the redistribution of electronic charge density resulting from interlayer sliding, which disrupts P or other crystalline symmetries such as the mirror symmetry $M_{z}$. Here, we consider a bilayer structure composed of two identical FM monolayers coupled in an A‐type compensated magnetic order throughout the sliding FE transition (see Figure 7a). In the NP phase, the spin sublattices of the upper and lower layers are linked by a combined symmetry operation [$C_{2}\left|\right|O$], where $C_{2}$ denotes the twofold rotation perpendicular to the spin axis, and O represents the transformation in real space (e.g., $M_{z}$). Consequently, this NP phase is identified as the NP AFM metal, characterized by the absence of out‐of‐plane polarization, nonrelativistic spin splitting, and net magnetization. Upon sliding away from the NP stacking toward either of the two polar FE stackings, the combined spin symmetry [$C_{2}\left|\right|O$] is broken, and the two spin sublattices cannot be connected by any symmetry operation. This symmetry breaking induces spin splitting in the band structure even in the absence of SOC (see Figure 7b). However, the macroscopic magnetic outcome diverges significantly depending on the electronic nature of the system:

- **Sliding FE Metals**: In the FE metallic phase, the presence of itinerant carriers at the Fermi level allows the spin‐split bands to generate unequal integrated DOS (iDOS) for the two spin channels below the Fermi level (i.e., $\mathrm{iDO}S^{↑}\left(E_{F}\right)\neq \mathrm{iDO}S^{↓}\left(E_{F}\right)$). Here, the iDOS is defined as
(1) $$iDOS^{\sigma }\left(E_{F}\right)=\int_{-\infty }^{E_{F}}D^{\sigma }\left(E\right)dE$$
  where *D* ^σ^(*E*) represents the spin‐resolved density of states, with σ  =  ↑ and ↓ denoting the spin‐up and spin‐down channels, respectively. The spin‐resolved iDOS below the Fermi level corresponds to the number of occupied electrons in each spin channel. According to the expression for the net magnetic moment **M** in a magnet, this imbalance produces a finite net magnetic moment. It can be written as
(2) $$M=\mu_{B}\left[iDOS^{↑}\left(E_{F}\right)-iDOS^{↓}\left(E_{F}\right)\right]$$
  where μ_B_ is the Bohr magneton and *E_F_* is the Fermi level. Thus, this phase is referred to as a sliding FE FiM metal, which exhibits the out‐of‐plane polarization, nonrelativistic spin splitting, and net magnetization. More importantly, the nonrelativistic spin splitting $\Delta E_{k}^{S}$ (denoted by **S**) could be reversed purely through the interlayer sliding FE transition (i.e., the reversal of the out‐of‐plane polarization (*P_z_*)), while the Néel vector remains unchanged. Based on Equation (2), the sign reversal of *S* further leads to reversal of the net magnetization (**M)**. As a result, the sliding FE FiM metallic system could enable triply‐coupled switching. Specifically, the initial FE‐FiM metal state $\psi_{k}^{i}\left(M,\;P_{z},\;S\right)$ and the final FE‐FiM metal state $\psi_{k}^{f}\left(-M,-P_{z},-S\right)$ are related by a combined operation [$C_{2}\left|\right|M_{z}$], and satisfy
(3) $$\left[C_{2}∥M_{z}\right]\psi_{k}^{i}\left(M_{N},P_{z},S\right)=\psi_{k}^{f}\;\left(-M_{N},-P_{z},-S\right).$$
- **Sliding FE Insulators/Semiconductors**: Conversely, in sliding FE insulators/semiconductors, the bandgap remains open throughout the sliding FE transition. Even after the symmetry connecting spin sublattices is broken, the occupied states below *E_F_* remain fully compensated (i.e., iDOS^↑^(*E_F_*)  = iDOS^↓^ (*E_F_*)). Consequently, the net magnetization remains zero, and such systems are referred to as FE fully compensated FiM insulators/semiconductors.

**FIGURE 7:**
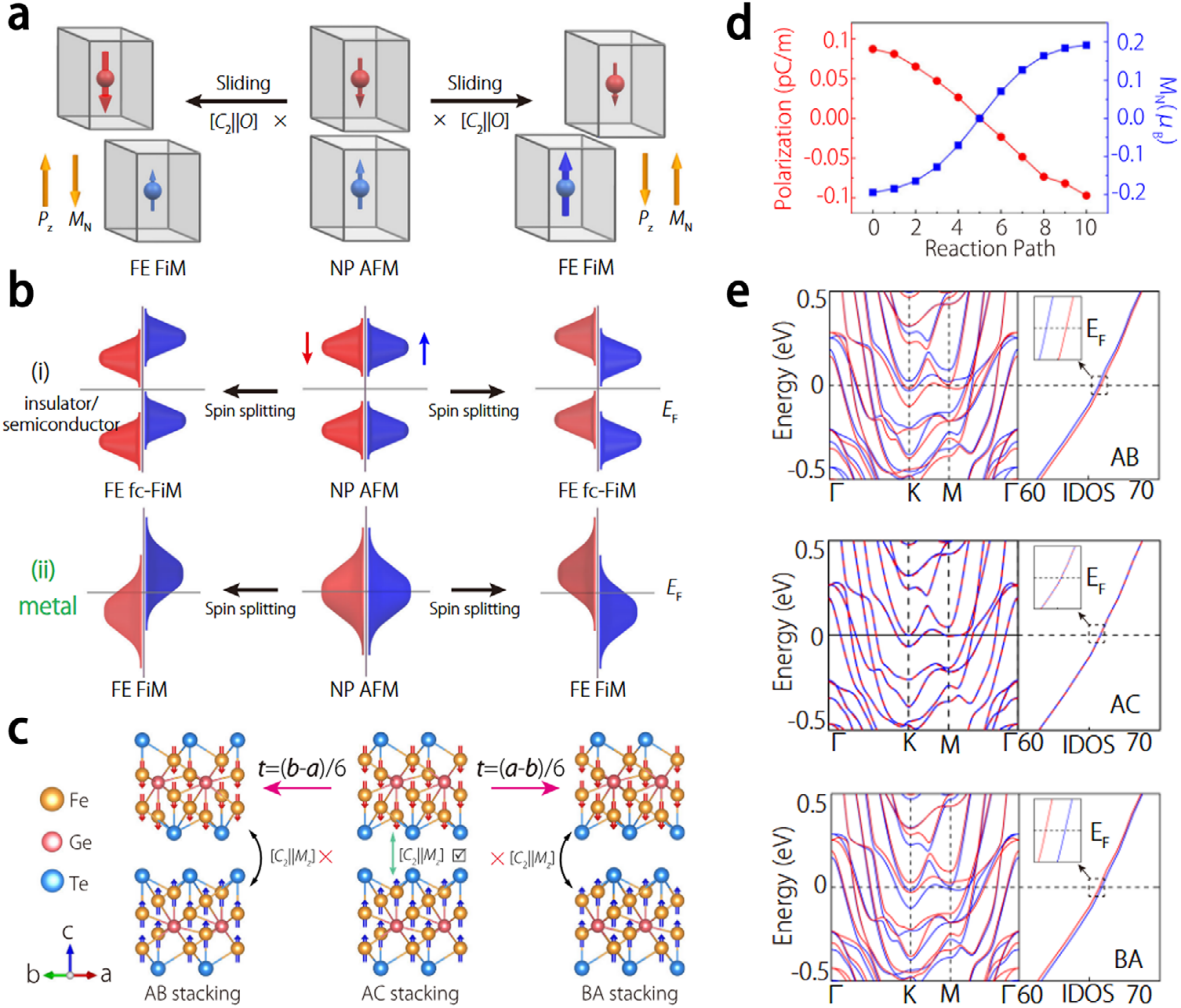
(a) Schematic illustration of the sliding FE transition from an NP AFM state to two FE FiM states with opposite polarization *P*_z_ and net magnetization **M**. (b) Evolution of spin‐resolved density of states across the sliding FE transition for (i) insulating/semiconducting and (ii) metallic systems, showing the emergence of spin splitting and net magnetization in the metallic case. (c) Structural evolution of the bilayer Fe_5_GeTe_2_ during interlayer sliding between AB, AC, and BA stackings. (d) Variation of the out‐of‐plane polarization *P*_z_ and net magnetic moment **M** along the sliding FE switching path. (e) Spin‐resolved band structures and iDOS for the AB, AC, and BA stacking configurations of the bilayer Fe_5_GeTe_2_. Reproduced with permission [69]. Copyright 2026, Springer Nature.

Building upon the symmetry framework, our recent study has identified a prototype material realization based on bilayer Fe_5_GeTe_2_, which exemplifies the symmetry‐breaking‐induced iDOS imbalance mechanism. As illustrated in Figure 7c, the system undergoes a structural transition from an NP AFM phase (AC stacking) to FE FiM phases (AB and BA stackings) driven by relative lateral displacement. The switching pathway reveals a low energy barrier of approximately 15.4 meV per unit cell. This modest barrier suggests energetic accessibility via external electric fields under practical conditions. The simultaneous evolution of out‐of‐plane polarization and net magnetic moment during the sliding process demonstrates robust coupled behavior. Specifically, *P_z_* reversal between the AB and BA states coincides with simultaneous switching of the net magnetization, whose magnitude reaches up to 0.19 µ_B_ per unit cell (see Figure 7d). The electronic origin of this coupling is elucidated in the band structure panels in Figure 7e. In the NP AC stacking, the preserved symmetry [$C_{2}\left|\right|M_{z}$] connects the opposite‐spin sublattices and enforces a spin‐degenerate AFM state in the absence of SOC. It ensures equal iDOS for spin‐up and spin‐down channels at the Fermi level, resulting in vanishing net magnetization. In contrast, for the FE AB and BA stackings, the symmetry breaking induces spin‐split bands that cross the Fermi energy. Due to the metallic nature of the system, the spin splitting leads to unequal iDOS values for opposing spin channels (i.e., $\mathrm{iDO}S^{↑}\left(E_{F}\right)\neq \mathrm{iDO}S^{↓}\left(E_{F}\right)$), thereby generating a finite macroscopic magnetic moment. In the absence of SOC, the two stackings of bilayer Fe_5_GeTe_2_ are related by the combined spin‐group operation [$C_{2}\left|\right|M_{z}$], under which the out‐of‐plane polarization, spin splitting, and net magnetic moment are simultaneously reversed, giving rise to triply‐coupled switching. A recent study on the bilayer MnIn_2_Se_4_ further established a general symmetry framework for sliding FE FiM metals and identified it as a topological sliding FE FiM metal hosting multiple Weyl points near the Fermi level. A tight‐binding Hamiltonian was further constructed to capture the microscopic mechanism underlying triply‐coupled switching [70]. This mechanism highlights how metallicity is essential for converting symmetry‐breaking spin splitting into a measurable net magnetization, distinguishing it from insulating counterparts where iDOS maintains a fully compensated magnetic state.

### 3.3 Application in Linear Magnetoelectric Response and Anomalous Transport

Beyond the realization of coexisting out‐of‐plane polarization, magnetism, and metallicity, magnetic sliding FE metals are particularly attractive because these coupled orders can be directly converted into functional responses. In such systems, external electric or magnetic fields can continuously tune the coupled polarization and magnetic degrees of freedom, leading to pronounced linear magnetoelectric coupling. At the same time, their metallic nature places electronic states directly at the Fermi level, which provides opportunities for pronounced Berry curvature and sizable anomalous transport responses. These responses could be non‐volatilely reversed under appropriate magnetic directions by interlayer sliding between opposite FE states. Such a combination of electrical switchability and enhanced anomalous transport response highlights the unique application potential of magnetic sliding FE metals.

This functionality is well illustrated by the bilayer 1*T*‐NbTe_2_, a sliding FE metal with A‐type compensated magnetic order [72]. As shown in Figure 8a–d, *1T*‐NbTe_2_ hosts a metallic electronic structure, an out‐of‐plane polarization of 0.34 pC/m, and a net magnetic moment of 0.066 μ_*B*_. The finite net moment is determined by incomplete interlayer compensation, and changes sign during the sliding FE transition. More importantly, the finite net magnetic moment is highly susceptible to external electric or magnetic fields, leading to substantial linear magnetoelectric coupling coefficients. The linear magnetoelectric coupling coefficients are conventionally defined by the relations μ_0_Δ*M*  = α^*E*^ *EV* and Δ*P*  = α^*H*^ *Hh*, where μ_0_ is the vacuum permeability, *V* is the unit cell volume, *h* is the effective thickness, and *E* and *H* represent the electric and magnetic fields, respectively. As shown in Figure 8e,f, the sliding 1*T*‐NbTe_2_ bilayer yields α^*E*^ =  1.41 ps/m and α^*H*^ =  1.46 ps/m. These values are comparable to those of the prototypical magnetoelectric material Cr_2_O_3_ (1.45 ps/m). Furthermore, Han et al. derived a universal formula for the magnetoelectric coupling parameters of multiferroic metals,

(4) $$\alpha^{E}=\frac{2\varepsilon_{0}\mu_{0}\mu_{B}}{e}\;\frac{\eta \kappa }{h}=C\frac{\eta \kappa }{h}$$

where ε_0_ and μ_0_ are the vacuum permittivity and permeability, μ_*B*_ is the Bohr magneton, *e* is the elementary charge, η is the spin polarizability of the transferred charge, κ is the interlayer dielectric permittivity, and *h* is the effective thickness. This expression indicates that the linear magnetoelectric response is an intrinsic feature in sliding multiferroic metals and is expected to exhibit substantial values.

**FIGURE 8:**
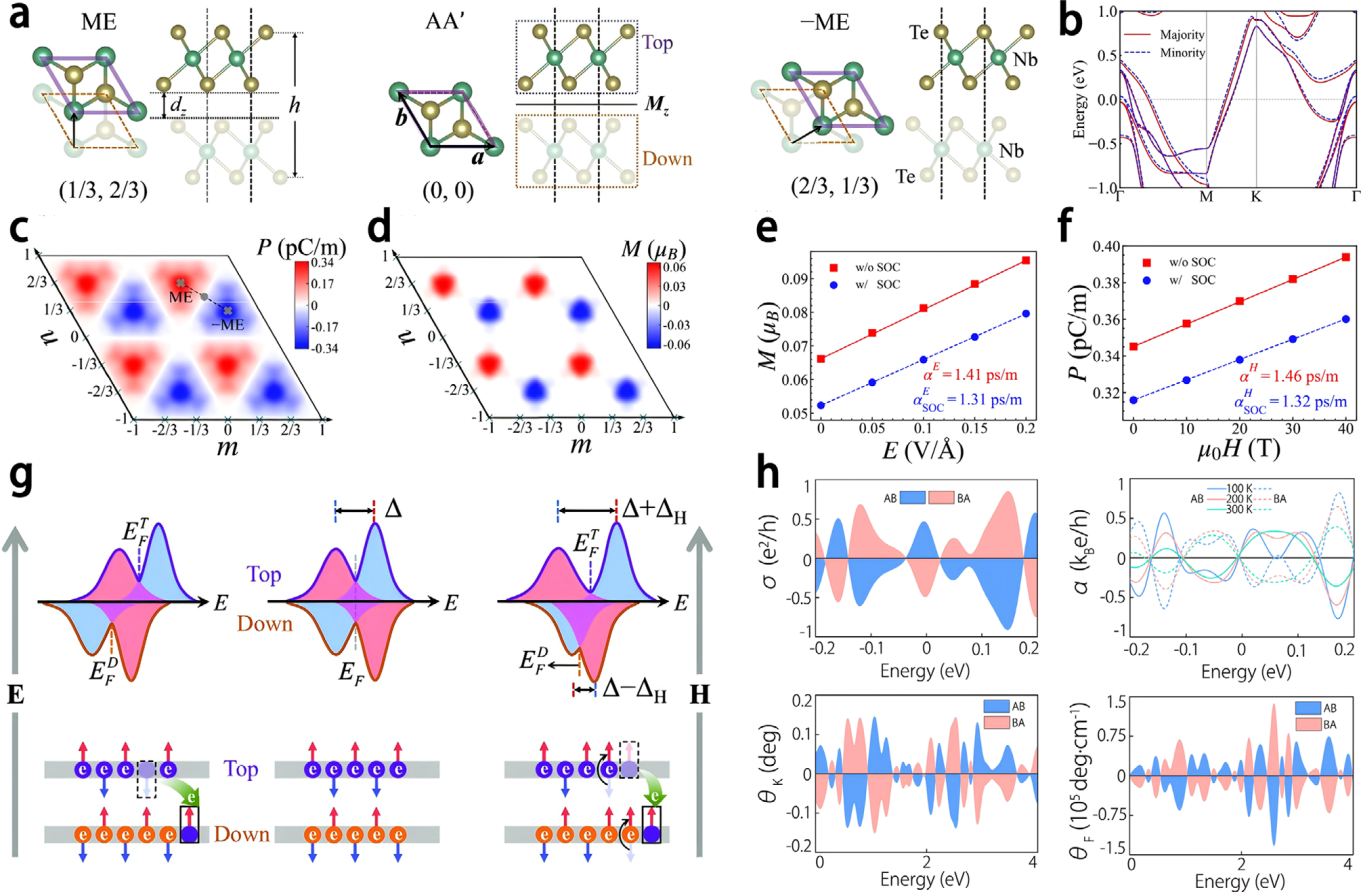
(a) Structural evolution of the bilayer 1*T*‐NbTe_2_ during interlayer sliding. (b) Spin‐resolved band structure of bilayer 1*T*‐NbTe_2_ showing metallic behavior with spin splitting. (c, d) Calculated out‐of‐plane polarization and total magnetic moment of bilayer 1*T*‐NbTe_2_ as functions of interlayer sliding. (e) Electric‐field dependence of the total magnetic moment with and without SOC. (f) Magnetic‐field dependence of the out‐of‐plane polarization with and without SOC. (g) Schematic illustration of the Fermi‐energy‐modulation mechanism underlying the magnetoelectric response. Reproduced under the terms of the CC‐BY 4.0 license [72]. Copyright 2026, The Authors, Published by American Physical Society. (h) Calculated anomalous Hall conductivity (upper left), anomalous Nernst conductivity (upper right), Kerr rotation angle (lower left), and Faraday rotation angle (lower right) for the AB and BA stackings of bilayer Fe_5_GeTe_2_. Reproduced with permission [69]. Copyright 2026, Springer Nature.

Microscopically, this behavior originates from a Fermi‐energy‐modulation mechanism identified in bilayer 1*T*‐NbTe_2_ by Han et al. Specifically, the applied vertical electric field induces a potential energy difference between layers, causing electronic states of one layer to shift relative to the other (see Figure 8g). To maintain an identical Fermi level across the system, electrons transfer between layers accompanied by spin flipping. This process prevents interlayer magnetic moments from fully compensating and modifies the total moment. Similarly, an applied magnetic field introduces Zeeman splitting, which broadens spin splitting in one layer while narrowing it in the other due to antiparallel interlayer coupling. Such asymmetry creates a difference in Fermi levels between layers, thereby driving interlayer charge transfer and altering the polarization. This represents a direct coupling between spin and charge degrees of freedom without relying on structural intermediaries or SOC.

Consistent with this general picture, bilayer Fe_5_GeTe_2_ also exhibits a pronounced linear magnetoelectric response. Estimating the coupling coefficient from the expression α ≈ μ_0_Δ*M*/*E* gives a value of about 7.13 × 10^−14^ G · cm^2^/V, which is markedly larger than those of conventional FM thin films and comparable to that of bilayer VS_2_ under a reduced interlayer spacing. Beyond this sizable linear magnetoelectricity, bilayer Fe_5_GeTe_2_ exhibits an additional functional advantage from its spin‐split metallic bands near the Fermi level, which enable pronounced anomalous transport and magneto‐optical responses. As shown in Figure 8h, in the FE phase of bilayer Fe_5_GeTe_2_, the anomalous Hall conductivity reaches 0.49 *e^2^/h* near the Fermi level, while the anomalous Nernst conductivity attains 0.36 *k_B_e/h* at 100 K and 0.34 *k_B_e/h* at 300 K—values exceeding those of prototypical noncollinear antiferromagnets. The magneto‐optical Kerr and Faraday rotations likewise reach 0.14 deg and 1.36 × 10^5^ deg/cm, respectively. Furthermore, when SOC is included, the symmetry connecting the AB and BA stackings depends on the Néel vector orientation. For an in‐plane Néel vector, they are related by $M_{z}$, under which σ_xy_ is even, leading to anomalous transport and magneto‐optical responses with the same signs. By contrast, for an out‐of‐plane Néel vector, the two states are related by $TM_{z}$, under which σ_xy_ is odd, leading to identical magnitudes but opposite signs for these responses (see Figure 8h). Notably, polarization control of anomalous Hall responses has previously been explored in several sliding FE insulating and semiconducting systems [106, 110, 111, 112, 113], such as bilayer MnBi_2_Te_4_ [114] and multilayers RuCl_2_ [115]. However, in the widely studied topologically trivial sliding FE semiconductors, the anomalous Hall conductivity vanishes when the Fermi level lies within the bandgap, and a finite response generally requires carrier doping. By contrast, sliding FE metals host metallic bands directly at the Fermi level, enabling sizable and electrically reversible anomalous Hall responses without extrinsic carrier doping and thereby offering promising platforms for low‐power spintronic and magneto‐optical applications.

## 4 Challenges and Prospects

This perspective systematically examines the emerging field of sliding FE metals, tracing their evolution from nonmagnetic prototypical systems to magnetic multifunctional platforms. We begin with the experimental realization and mechanistic understanding of nonmagnetic sliding FE metals in 1T′‐WTe_2_ and related systems, highlighting their unique interlayer charge transfer origin and the resulting Berry curvature memory and electrically switchable superconductivity. Furthermore, within a symmetry‐based framework for integrating compensated magnetic orders into sliding FE metals, we then reveal that the transition from NP AFM to FE FiM phases enables triply‐coupled switching—simultaneous reversal of FE polarization, spin splitting, and net magnetization without altering the Néel vector. In addition, these systems display sizable linear magnetoelectric coupling coefficients together with electrically tunable anomalous transport properties, including sign‐reversible anomalous Hall conductivity, anomalous Nernst effect, and magneto‐optical responses. These developments highlight the substantial potential of sliding FE metals for multifunctional spintronic and quantum devices.

Despite the rapid progress in this field in recent years, several critical challenges remain to be addressed before sliding FE metals can realize their full potential for practical applications:

- Limited material database. To date, experimentally confirmed sliding FE metals remain scarce and are predominantly confined to nonmagnetic systems such as 1T′‐WTe_2_ and T_d_‐MoTe_2_. The exploration of magnetic sliding FE metals is still in its infancy, with only a few theoretical predictions available. To overcome this limitation, future efforts should focus on high‐throughput first‐principles screening of experimentally synthesizable monolayer magnetic metals and on constructing bilayer and multilayer heterostructures with sliding FE capability. Key screening criteria should include high Curie/Néel temperature, low switching barrier, and sizable coupling between polarization and magnetization, thereby establishing a comprehensive material database for magnetic sliding FE metals.
- Topological quantum states. While nonmagnetic sliding FE metals have demonstrated Berry curvature memory effects, the exploration of topological quantum states in magnetic sliding FE metals remains largely uncharted. The interplay between nonrelativistic spin splitting arising from compensated magnetic orders and sliding‐induced FE switching could potentially enable electrically switchable topological phases. Identifying realistic material platforms that harbor such topological states and enable electric‐field control of their topological invariants represents a compelling direction for future theoretical and computational investigation.
- Experimental verification of magnetic sliding FE metals. Despite compelling theoretical predictions, the experimental realization of magnetic sliding FE metals has yet to be achieved. In particular, artificially stacked van der Waals multilayers combining metallicity, electrically switchable polarization, and magnetism remain theoretical proposals awaiting experimental validation. The primary challenge lies in the precise control of stacking configurations in magnetic vdW multilayers and the unambiguous characterization of both FE polarization and magnetic order in the same device. Advanced fabrication techniques, such as tear‐and‐stack assembly with crystallographic alignment, combined with local probe measurements including piezoresponse force microscopy and magnetic circular dichroism, will be essential to validate the coexistence and coupling of ferroelectricity and magnetism in these systems.
- Domain formation and moiré effects. In realistic vdW‐assembled polar heterostructures, small twist angles, lattice mismatch, and local strain can generate moiré patterns and drive lattice reconstruction into domains with alternating polarization separated by domain‐wall networks [59, 116, 117, 118, 119]. This issue could be particularly consequential in magnetic sliding FE metals because opposite polar states may host reversed magnetization and anomalous transport responses. Potential mitigation strategies include achieving fully commensurate stacking [120], using nearly lattice‐matched constituents to form untwisted and commensurate interfaces [57], employing vapor–liquid–solid growth to obtain uniform single‐domain structures [121], and using engineered boundary confinement to alleviate domain‐wall pinning and facilitate non‐volatile polarization switching [122].
- Direct demonstration of triply‐coupled switching and transport phenomena. Even once magnetic sliding FE metals are experimentally realized, the direct observation of triply‐coupled switching—simultaneous reversal of out‐of‐plane polarization, net magnetization, and spin splitting—remains a formidable challenge. Magnetotransport and magneto‐optical measurements, including the anomalous Hall effect and magneto‐optical Kerr and Faraday effects, in dual‐gate devices will be crucial to establish the correlation between FE switching and magnetic response reversal. Furthermore, the predicted sizable linear magnetoelectric coefficients and electrically controllable anomalous transport properties await experimental confirmation, which would provide decisive evidence for the underlying coupling mechanisms.
- Device‐oriented functionality exploration. Beyond fundamental characterization, the potential of sliding FE metals for practical devices remains largely unexplored. Inspired by the Berry curvature memory demonstrated in nonmagnetic WTe_2_, magnetic sliding FE metals could enable non‐volatile electrical control of Berry curvature‐related phenomena, such as the anomalous Hall effect and nonlinear Hall effect, with potential applications in low‐power magnetic memories. Exploring these device geometries through both theoretical modeling and experimental implementation will be essential to translate the unique properties of sliding FE metals into functional spintronic technologies.

Addressing these challenges will require concerted efforts across theory, materials synthesis, and device fabrication. The rich interplay between sliding ferroelectricity, magnetism, metallicity, and topology promises not only to deepen our understanding of quantum materials but also to unlock new paradigms for energy‐efficient electronics and spintronics.

## Author Contributions

**Ying Yang**: writing – original draft, funding acquisition, writing – review and editing. **Zhenzhou Guo**: writing – original draft, writing – review and editing, supervision. **Xia Cheng**: writing – original draft. **Xiaotian Wang**: funding acquisition, supervision, writing – review and editing.

## Conflicts of Interest

The authors declare no conflicts of interest.

## Data Availability

No new data were generated in this Perspective.
